# Resolution and contrast enhancement of subtractive second harmonic generation microscopy with a circularly polarized vortex beam

**DOI:** 10.1038/srep13580

**Published:** 2015-09-14

**Authors:** Nian Tian, Ling Fu, Min Gu

**Affiliations:** 1 Britton Chance Center for Biomedical Photonics, Wuhan National Laboratory for Optoelectronics, Huazhong University of Science and Technology, Wuhan 430074, China; 2 Key Laboratory of Biomedical Photonics of Ministry of Education, Department of Biomedical Engineering, Huazhong University of Science and Technology, Wuhan 430074, China; 3 Centre for Micro-Photonics, Faculty of Science, Engineering and Technology, Swinburne University of Technology, Hawthorn, Victoria 3122, Australia

## Abstract

We extend the subtractive imaging method to label-free second harmonic generation (SHG) microscopy to enhance the spatial resolution and contrast. This method is based on the intensity difference between two images obtained with circularly polarized Gaussian and doughnut-shaped beams, respectively. By characterizing the intensity and polarization distributions of the two focused beams, we verify the feasibility of the subtractive imaging method in polarization dependent SHG microscopy. The resolution and contrast enhancement in different biological samples is demonstrated. This work will open a new avenue for the applications of SHG microscopy in biomedical research.

Second harmonic generation (SHG) is related to the molecular orientation on a submicrometer scale. SHG microscopy, with its unique imaging properties, has been gaining much interest and popularity as a method to study well-structured samples without the need for fluorescent probes^1^,^2^,^3^,^4^,^5^,^6^,^7^. As with fluorescence microscopy techniques, there is a growing demand for enhancing spatial resolution to see more details. Recent years have seen the development of novel techniques for super-resolution beyond the diffraction limit. State-of-the-art techniques, including stimulated emission depletion (STED)^8^, molecular photoactivation^9^,^10^ and structured illumination^11^ enable the significantly enhanced resolution in biological imaging. Among these approaches, structured illumination is combined with widefield microscopy, while STED and the molecular photoactivation methods rely on specific photo-physical properties of fluorescent probes. However, SHG microscopy is based on the nonlinear optical effect of SHG and obtain its contrast from variations in a specimen’s ability (second-order nonlinear susceptibility) to generate SH light from the incident light. Thus these fluorescence or reflectance superresolution imaging techniques can not be retrofitted in SHG microscopy which holds a different contrast mechanism.

Other approaches based on the beam engineering techniques are proposed to overcome the limitation of requirement for specific fluorescent probes in the afore-mentioned methods. A direct way to achieve the resolution enhancement is to obtain a sharper focal spot for scanning^12^,^13^. Some studies have introduced this kind of beams into the conventional laser scanning microscopy and enabled the lateral resolution enhancement^14^. Nevertheless, the sharper focal spot is produced at the cost of side lobes and the elongation in the axial direction, which leads to contrast and axial resolution degradation in imaging.

An alternative and less direct strategy for the resolution enhancement is based on the intensity subtraction between two scanned images under the illumination of a solid focal spot (bright spot) and a doughnut-shaped hollow focal spot (dark spot). This method exploits the smaller feature size of the dark spot and its validity has been well demonstrated in confocal and two-photon excitation fluorescence (TPEF) microscopy^15^,^16^,^17^. Unlike two-photon fluorescence excitation, SHG excitation is a second-order nonlinear process and the signal field is directly proportional to the conjugated product of the excitation field^18^. The coherent emission feature of SHG determines the polarization dependence of the harmonic light. The observed SH intensity is highly dependent on the angle between the molecular orientation and the polarization direction of the exciting field^1^,^6^,^18^. So besides the intensity distributions, we must consider the polarization distributions of the exciting beams in the focal region. The properties of the intensity dependency in SHG microscopy make it necessary for the two different exciting light fields to have the same polarization at the corresponding points to ensure that the point by point subtractive scheme is still valid. Therefore, additional considerations on the control of the polarization states in the beam engineering process are indispensable and a pair of bright and dark beams with matched intensity and polarization distributions are essentially needed if we apply subtractive imaging to SHG microscopy. So far, the combination of subtractive imaging with polarization dependent SHG microscopy is still pending and has not been investigated.

In this paper, we use a circularly polarized beam as the bright beam. An apodized vortex phase modulated circularly polarized beam is used as the dark beam to form a doughnut-shaped intensity distribution in the focal field. We characterize the polarization distributions of the bright and dark focal fields formed by these two beams to verify their polarization homogeneity. Taking advantage of the flexibility of dynamic diffractive optical elements, we further implement the subtraction method in SHG microscopy to demonstrate the resolution and contrast enhancement in the SHG microscopy images.

## Results

The generation of the bright and dark focal spots is critical in the subtractive imaging. Supplementary Fig. S1 lists the intensity and polarization distributions of the common bright and dark focal spots. A circularly polarized and a circularly polarized vortex beam may satisfy the requirements of the bright and dark beams for SHG imaging (see Supplementary Information for details). On the other hand, SH excitation has the effects of broadening the dark central zone of the dark beam since SH signal intensity scales as the square of excitation intensity. This will diminish the ability of resolution enhancement and introduce more negative values in the subtraction, which can cause severe deformations^19^,^20^. To overcome these disadvantages, we employ an apodized circularly polarized vortex beam to reduce the dimension of the dark area at the center of the dark beam.

**Figure 1:**
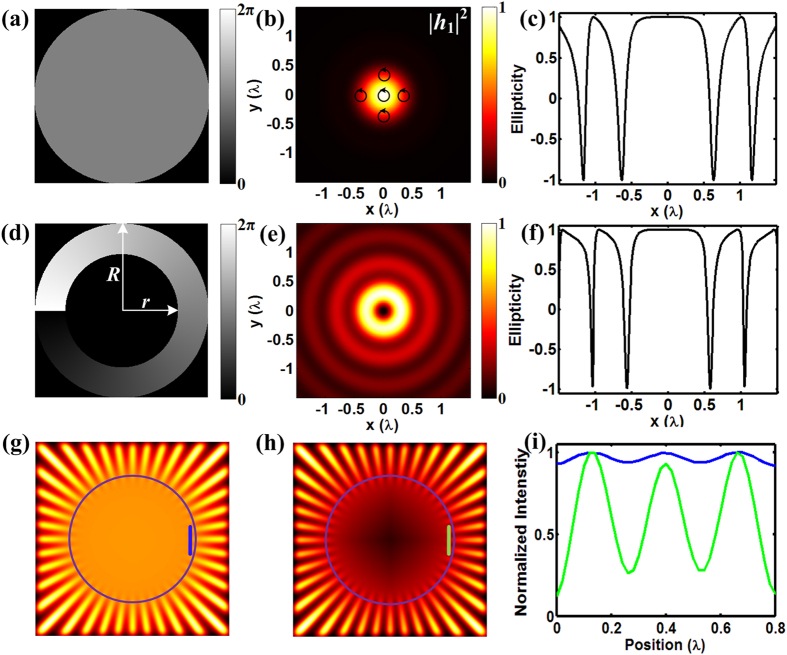
(**a**,**d**) generated phase function for the bright and dark beam. The apodization factor ε = *r*/*R* = 0.65. (**b**,**e**) Corresponding intensity distribution in the focal region for the phase pattern of (**a**,**d**) respectively. (**c**,**f**) A cross section of the ellipticity of the focused bright (**b**) and dark (**e**) beams. (**g**–**i**) Simulated images of a “star-like” test sample. (**g**) Conventional image. (**h**) Subtractive image with a subtractive factor γ = 0.6. (**i**) Comparison of the intensity profiles along the lines in conventional (blue) and subtractive (green) images.

The designed phase modulation function and the corresponding intensity and polarization distributions in the focal region are depicted in Fig. 1(a,b,d,e), respectively, a constant phase for the bright beam illumination and a vortex phase for the dark beam illumination. For the apodization, the center area of the modulated beam is blocked. This can be simply achieved by adding an annular blazed diffraction grating to the vortex phase pattern (not shown in Fig. 1(d)). The annular blazed diffraction grating acts as an annular pupil through shifting the beam illuminating on it to the first order to form an annular beam. Intensity distributions under high numerical-aperture (NA) objective (1.1 NA, 60×) are calculated by using the Debye diffraction theory^21^.

In SHG microscopy, a high NA objective is typically used to focus the excitation beam, and the SH intensity is highly dependent on the polarization state of the exciting field. However, a high NA objective may cause depolarization when a beam is focused^21^. Therefore it is necessary to examine the polarization distribution of the light field in the focal plane. Since the transversal components still dominate in the focal fields (see Supplementary Information for details), we only characterize the transverse polarizations. This is always done by calculating the Stokes parameters of the field at each point, from which the ellipticity tanχ of the polarization ellipse can be derived as^22^,^23^


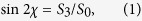


where *S*_i_ denotes the Stokes polarization component exploited to describe the polarization state. Since the light fields are symmetrically distributed with respect to the axis, only a cross section of the ellipticity is presented for clarity. Fig. 1(c) shows the ellipticity of the bright light field. It can be seen that the polarization keeps left-hand circularly polarized and have no apparent changes in the main lobe where most of the energy concentrates. For the dark light field whose ellipticity is shown in Fig. 1(f), the polarization is also left-hand circularly polarized and keeps almost invariant at the ‘doughnut’, whose zero intensity is in the center and the peak locates in the outer ring.

To be noted, accompanied with the intensity decreasing, the ellipticity drops sharply and the polarization state changes rapidly at the boundaries of the two light fields. However, second harmonic generation is a second-order nonlinear optical process and the signal scales as the square of the intensity^18^, second harmonic only produces within the focal area near the peak intensity. Thus the periphery of the focused field contributes less to excite the SH signal due to lower energy density. While the apodization reduces the size of the dark spot, it also causes side lobes, as can be seen from Fig. 1(c). However, the nonlinear effect of the SH excitation can weaken the intensity of the side lobes. So we can conclude that the efficient exciting fields using circularly polarized and circularly polarized vortex beam have homogeneous circular polarizations at each point. This forms the basis of the application of point-by-point subtractive method to SHG microscopy that is highly polarization-dependent.

Our method also takes two separate scanned images under the illumination of the above two focal spots for the subsequent subtraction, which is similar to that used in confocal and two-photon fluorescence subtractive imaging^15^,^16^,^17^. However, the image formation in SHG microscopy is different from that in the fluorescence case. Physically, fluorescence microscopy and SHG microscopy correspond to incoherent and coherent imaging processes, respectively. As SHG is a coherent process, the imaging process in SHG microscopy cannot be described by an optical transfer function^24^,^25^. Thus the description of the resolution and contrast enhancement in fluorescent subtractive imaging is not applicable to our subtractive SHG microscopy. In order to investigate the imaging performance of subtractive SHG microscopy we describe the final subtractive image based on the coherent image formation process (see details in Method).

To evaluate the power of resolution enhancement in our SHG case, we perform a simulation test on a two dimensional “star-like” sample^26^. The object function of the sample is given by *O*_*e*_(*r*, *θ*) = 1 + cos(40*θ*), where (*r*, *θ*) are the polar coordinates in the sample plane. The radial features are more difficult to be discerned when one moves closer to the image center and there always exists a limit radius under which they are unresolved. Thus this sample is ideal to be employed to study the performance of imaging techniques since the resolution can be accessed simply by measuring the limit radius^16^. We can obtain the subtractive SHG image of the sample by substituting the object function to Equation (1) in the Method while the first term of the equation exactly represents the conventional SHG image under the scanning of the Gaussian bright focal spot. The conventional and subtractive images are shown in Fig. 1(g,h), respectively. According to the Rayleigh criterion, two peaks can be resolved when ratio of the valley and peak intensity values is less than 73.5%. The resolution accessing from the limit radius is 0.35λ for the conventional image and 0.26λ for the subtractive image, indicating an improvement of 26% in the resolving ability. The purple circle in Fig. 1(g,h) denotes the resolution limit of the conventional image. In the conventional image, the features in the region inside the circle can hardly be discerned while some details in the same region can be clearly resolved. This result verifies the feasibility of subtractive imaging in our SHG case to enhance the resolution. Fig. 1(i) shows the comparison of the intensity profiles indicated by the blue and green lines in the images, which are very close to the purple circle. While in the conventional case the valley intensity is higher than 73.5% of the peak value, the valley intensity in the subtractive image is below this threshold. This result further demonstrates the resolution and contrast improvement.

A spatial light modulator (SLM) is used for wavefront shaping to generate the desirable bright and dark beams. Supplementary Fig. S4 outlines the experimental setup for subtractive SHG microscopy, which is constructed by integrating a SLM to a home built multiphoton microscope (see Method for details). To verify the formation of the two beams in the focal field by using the two phase modulations depicted in Fig. 1(a,d), we place a mirror in the focal plane of the objective and record the reflected images with a beam analyzer (Thorlabs, not shown in Supplementary Fig. S4). The intensity distributions of the two beams are shown in Supplementary Figs S5(a) and S5(b). The theoretical (solid lines) and experimental (dotted lines) intensity profiles along the line across the center of the focused fields are depicted in Supplementary Fig. S5(c), in which blue line is for the bright beams and red line is for the doughnut-shape dark beam. It can be seen that the experimental measurements of the intensity distributions of the two beams fit well with theoretical calculations.

**Figure 2:**
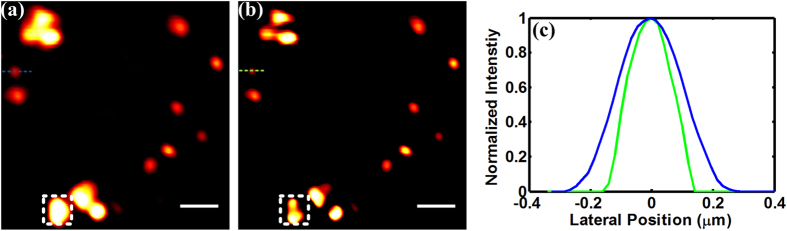
Conventional (**a**) and subtractive SHG images (**b**) of the BaTiO_3_ nanoparticles. (**c**) Intensity profiles along the dash measurement lines in (**a**,**b**). Scale bar: 1 μm.

With the above analysis and formed focused fields, we implement the subtractive imaging method in SHG microscopy. To illustrate the performance of resolution enhancement in SHG microscopy, we implement this method on BaTiO_3_ nanoparticles (NPs). This kind of NPs lack the central symmetry points and have high second order nonlinear susceptibilities, which means they are capable of generating SH signal. The sizes of NPs and the small aggregates rang from one hundred to a few hundred nanometers. Thus the NPs are ideal samples to measure the resolution achievable in SHG microscopy. We scan the sample with the two beams to obtain two images. The resulting subtractive image is constructed by the subtraction of the two scanned images. Fig. 2(a,b) show the conventional and subtractive SHG images respectively. It can be seen that the aggregated NPs, as indicated in the white boxes, are easier to be distinguished in the subtractive image. The comparison of the intensity profiles of a single NP in Fig. 2(c) shows the reduction of the measured size. Specifically, the full width at half maximum (FWHM) of the blue line profile is 280 nm while the FWHM of the green line profile is 210 nm. The result shows that a 25% improvement in the resolution is achieved in subtractive image.

**Figure 3:**
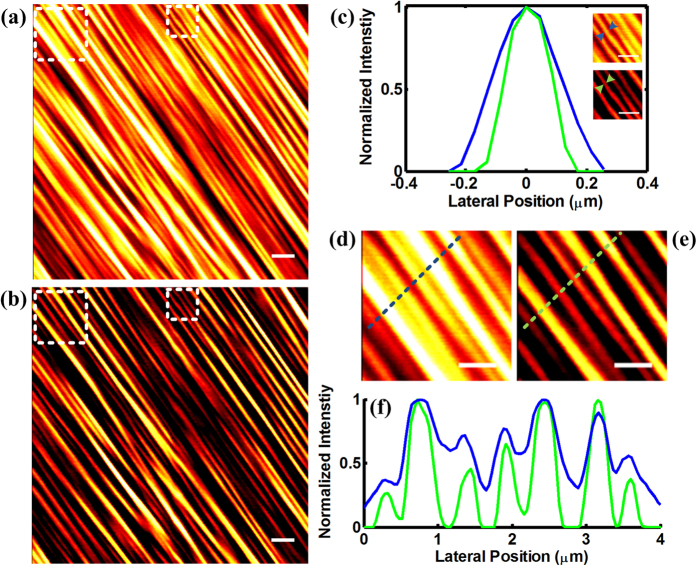
SHG images of a rat tail tendon with conventional SHG microscopy (**a**) and subtractive method (**b**). (**c**) Intensity characterization of the resolution enhancement. (**d**,**e**) magnified region of (**a**,**b**). (**f**) Intensity profile along the dashed line in (**d**,**e**). Scale bar: 1 μm.

Next we demonstrate the ability of this technique to enhance resolution in biological tissue samples by imaging rat tendons with fine structures^27^. The conventional and subtractive SHG images are shown in Fig. 3(a,b), respectively. It is clear that clusters of fibers presented in the conventional image are well differentiated by subtractive imaging. To evaluate the performance of the resolution enhancement, we measure the width of a single fiber. The insets in Fig. 3(c) correspond to the areas in the small white boxes in Fig. 3(a,b). The intensity profiles of the fiber indicated by the arrowheads in the inset are plotted. Obviously, the width of the fiber in subtractive image reduces sharply and the resolution is substantially enhanced. The resolution enhancement is much clearer when the images are enlarged. Fig. 3(d,e) are enlarged views of the region indicated by the big white boxes in Fig. 3(a,b), respectively. We plot the intensity along the measurement line, which is in the direction perpendicular to the orientation of the fibers, as shown in Fig. 3(f). It can be seen that the peaks corresponding to the fibers are resolved clearly with subtractive imaging while some of the closely packed peaks cannot be distinguished. In addition to resolution improvement, enhancement in contrast is also achieved as can be determined from the ratio of the peak and valley intensity values. Thus we demonstrate the resolution and contrast enhancement in SHG microscopy with the subtractive imaging method.

As discussed above, different from fluorescence microscopy, the SHG microscopy method is highly polarization-dependent. However, since our exciting fields are both circularly polarized, they can excite fibrils with all orientations equally. Thus our method has no constraints on the orientation of the samples. To demonstrate this property, we image different regions of the sample where the fibrils are in different directions. Supplementary Figs S6(a) and S6(c) show the conventional SHG images of the collagen fibrils whose longitudinal axis is along the vertical and horizontal directions respectively. Supplementary Figs S6(b) and S6(d) are the corresponding subtractive images. A comparison of the upper and lower panels reveals that the subtractive imaging is more powerful in differentiating the fibrils than that of the conventional imaging in both cases. Resolution enhancement is achieved regardless of the orientations of the fibrils. Thus we can conclude that this method does not depend on the orientation of the sample.

SHG microscopy is an effective, minimally invasive imaging modality in biomedical sciences and it can provide structure information that is absent from fluorescence imaging alone in specific applications. As our method is complementary to the fluorescence resolution enhancement techniques, it makes SHG microscopy a more powerful tool in biomedical studies. SHG microscopy is always used to visualize defects in muscles without need for exogenous fluorophores and complex sample preparation^28^,^29^. To demonstrate improvement of the image quality to detect muscle defects, we apply our method to image the mouse skeletal muscle myofibrils. Skeletal muscle fibres have a regular, periodic organization^30^. The repeat units, sarcomeres, are the structural basis for producing the force responsible for contractile function. In a healthy muscle, sarcomeres are rarely interrupted and are in register between several neighboring fibres.

**Figure 4:**
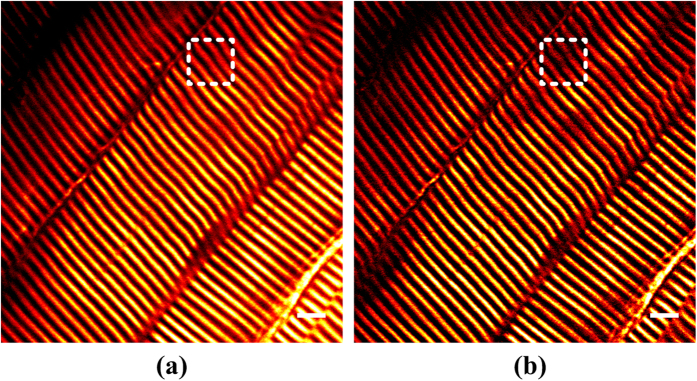
Conventional (**a**) and subtractive (**b**) SHG images of mouse leg muscle myofibrils. Scale bar: 5 μm.

Figure 4 shows the conventional and subtractive SHG images of mouse leg muscle myofibrils. We can see that the muscle has large fibres with regular striations of even spacing. However, small defects interrupting the striations still exist, such as those in the white boxes in the conventional image, though not so obvious. But they are very evident in the subtractive image, as shown in the corresponding boxes. Due to the improved contrast and resolution in the SHG image, the method is more helpful to assess muscle conditions.

## Discussion

Since a dynamic diffractive element is used in our method, we can realize the bright and dark modes and acquire the two images in only one optical path just by changing the phase pattern for SLM. There is no need to use a mechanical rotator to switch between the two modes^15^ and a complicated overlapping of two beams^16^ is also dispensable. This makes our system to be easily retrofitted in commercial microscopes.

In this study, we have implemented the subtractive imaging method in polarization dependent SHG microscopy. The resolution and contrast enhancement was demonstrated in different kinds of samples. A resolution improvement with almost 1.3 folds was achieved and a significant contrast enhancement was obtained without any priori information of samples. Furthermore, this method can also be implemented in two-photon microscopy with the same setup. The combination of TPEF from intrinsic fluorophores and SHG from endogenous structural proteins has been used widely for high-resolution and *in vivo* structural and functional imaging^31^,^32^. The method in this paper can be easily integrated to multimodal imaging platform and will be a powerful tool in nonlinear microscopy for non-invasive *in vivo* imaging in biomedical studies.

## Method

### Image formation of subtractive SHG microscopy

Assuming that the electrical field of the SHG emission from a sample is proportional to the square of the total illumination field on the sample^24^, the detected SH image can be expressed as 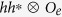, where ^*^ denote the conjugate operation and 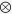 denotes the 2-D convolution operation, *h* is the 2-D amplitude point spread function for the illumination objective and *O*_e_ is the object function representing the SHG strength of the object.

The conventional image obtained using a Gaussian beam is denoted as *I*_*g*_ while the image acquired using doughnut-shaped beam is denoted by *I*_*d*_. The final subtractive image is constructed by the intensity subtraction of the two images after normalization. A weighing factor γ is introduced in the subtraction procedure *I*_*s*_ = *I*_*g*_ − γ*I*_*d*_. Considering the consistency of the polarization of the two exciting fields, the difference SHG image can be described as^33^


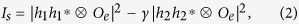


where *h*_1_ and *h*_2_ are the amplitude point spread functions under the illumination of the bright and dark beams respectively, as show in Fig. 1. The operation of subtraction inevitably creates negative values which are always set to zeros. However, large negative values may deteriorate the quality of the original information. Thus the weighing factor must be carefully selected to avoid exaggerated contrast enhancement (see Supplementary information for details). We use a factor around 0.5 in our experiments and no obvious artifacts are found compared with the original images.

### Experimental setup

Supplementary Fig. S4 outlines the experimental setup. A mode-locked femtosecond pulsed laser (MaiTai, 100 fs, 80 MHz, Spectra-Physics) is used for the generation of the second harmonic signal. The linearly polarized beam from the laser is illuminated on a SLM (1920 × 1080 pixels, 256 gray levels, Pluto-NIR, Holoeye Photonics AG) after being expanded to match the dimensions of the phase display. A 4*f* system relays the modulated light to an XY scanner. A spatial filter is placed at the intermediate Fourier plane of the 4*f* system to block the undiffracted zeroth order. The scanner is composed of a pair of galvanometers (X and Y, 6-mm beam aperture, model 6215H, Cambridge Technology Inc.) for eventually two-dimensional raster scanning. The galvanometers are made optically conjugate to the SLM. Another 4*f* system serves to conjugate the galvanometers to the back aperture of the objective (40×/0.95NA or 60×/1.1NA, USI-UPLAPO, Olympus). The ultimate conjugation of the SLM to the objective rear pupil is critical in order to generate the desirable light field in the focal plane. Before the light goes through the objective, a quarter wave plate is used to convert the linear polarization into the circular polarization. In the detection path, a long-pass dichroic beamsplitter (FF665-Di02-25 × 36, Semrock) above the objective transmits the excitation light and diverts the generated SH signal to a photomultiplier tube (PMT, R3896, Hamamatsu). A narrow band filter (FF01-390/15, Semrock) is placed before the PMT to isolate the SH signal from the fundamental and any fluorescence.

Actually, the phase patterns in Fig. 1(a,d) are added with a blazed diffraction grating to preferentially diffract light into the positive first order. In our experiments, we adjust the power of the bright and dark beams to ensure that the maximum intensities in *I*_*g*_ and *I*_*d*_ are similar.

### Sample preparations

The purchased NPs (Barium Titanate (BaTiO_3_), Sigma-Aldrich) were dispersed in ethanol and deposited on to a glass microscope cover slip and dried. The sample was stored in the drying oven until right before experiments. Rat tails in this study came from an 8-week-old mouse and were obtained immediately after the rat was sacrificed for other study. The tendon was cut and immersed in phosphate-buffered saline solution and then placed on a glass slide and sealed with a cover glass. The mouse leg muscle specimen came from another 8-week-old mouse after sacrifice for other study and the sample preparation was similar as that for rat tail tendon. All animal studies were performed in compliance with protocols that had been approved by the Hubei Provincial Animal Care and Use Committee and with the experimental guidelines of the Animal Experimentation Ethics Committee of Huazhong University of Science and Technology.

## Additional Information

**How to cite this article**: Tian, N. *et al.* Resolution and contrast enhancement of subtractive second harmonic generation microscopy with a circularly polarized vortex beam. *Sci. Rep.* **5**, 13580; doi: 10.1038/srep13580 (2015).

## Supplementary Material

Supplementary Information
