# Supplementary material for: Resolution and contrast enhancement of subtractive second harmonic generation microscopy with a circularly polarized vortex beam
Source: Sci Rep. 2015 Sep 14;5:13580. doi: 10.1038/srep13580 (PMC4568467; doi:10.1038/srep13580)
Supplement: Supplementary Information [file srep13580-s1.pdf]

Supplementary Information for
Resolution and contrast enhancement of subtractive second harmonic generation microscopy with a circularly polarized vortex beam

Nian Tian[1,2], Ling Fu [1,2*], Min Gu[3]

[1]Britton Chance Center for Biomedical Photonics, Wuhan National Laboratory for Optoelectronics, Huazhong University of Science and Technology, Wuhan 430074, China

[2]Key Laboratory of Biomedical Photonics of Ministry of Education, Department of Biomedical Engineering, Huazhong University of Science and Technology, Wuhan 430074, China

[3] Centre for Micro-Photonics and CUDOS, Faculty of Engineering and Industrial Sciences, Swinburne University of Technology, Hawthorn, Victoria 3122, Australia

Corresponding Author: **lfu@mail.hust.edu.cn*

S1. Polarization and intensity characterization of the bright and dark beams

Supplementary Fig. S1 lists the intensity and polarization distributions of the common bright and dark focal spots. The focused fields of linearly and circularly polarized beams form a solid spot and almost maintain the polarizations despite of the depolarization effect. Meanwhile, the tightly focused radially polarized beam shows a strong longitudinal field at its center and the transverse field presents radially polarized.[1] Generally two main families of dark beams are employed in imaging, namely the azimuthally polarized beam[1] and the circularly polarized vortex beam[2]. For the azimuthally polarized beam, the focused field is purely transverse and azimuthally polarized. It is hard to find a solid bright spot to have the matched polarizations. Fortunately, circular polarization still dominates in the focal field when a vortex 0–2π phase is used to modulate the circularly polarized beam. Therefore, a

circularly polarized and a circularly polarized vortex beam may satisfy the requirements of the bright and dark beams for SHG imaging.

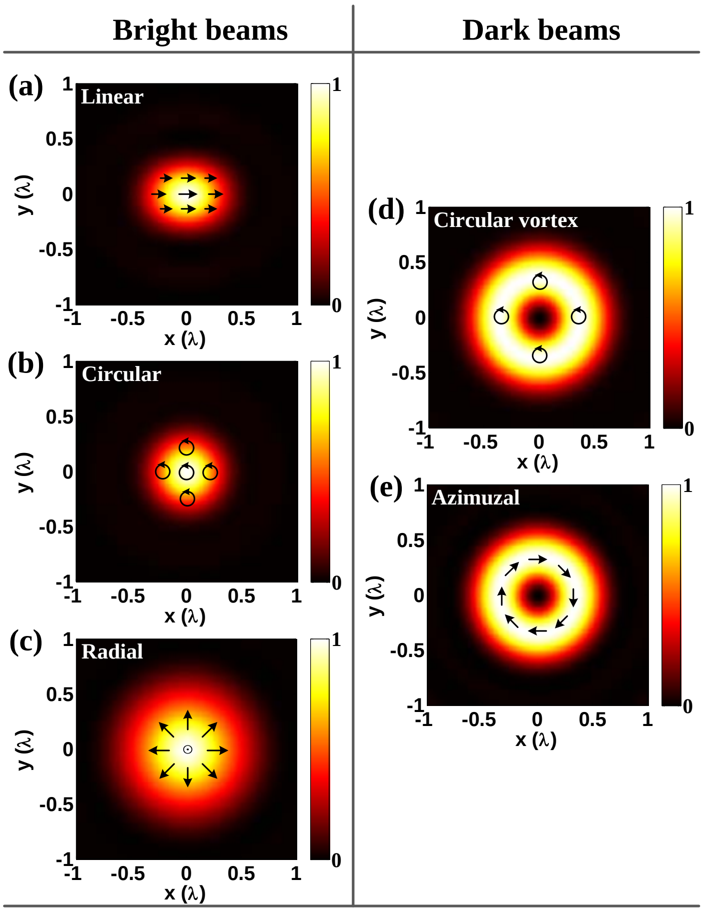

Fig. S1 Intensity and polarization distributions of the common bright and dark focal spots in the focal plane. Bright spots: various polarizations are considered, linear (a), circular (b) and radial (c). Dark spot: focused fields of circularly polarized beam with a vortex 0–2π phase modulation (d) and azimuthally polarized beam (e). The calculation was done with a high-NA water-immersion objective (1.1 NA, 60×).

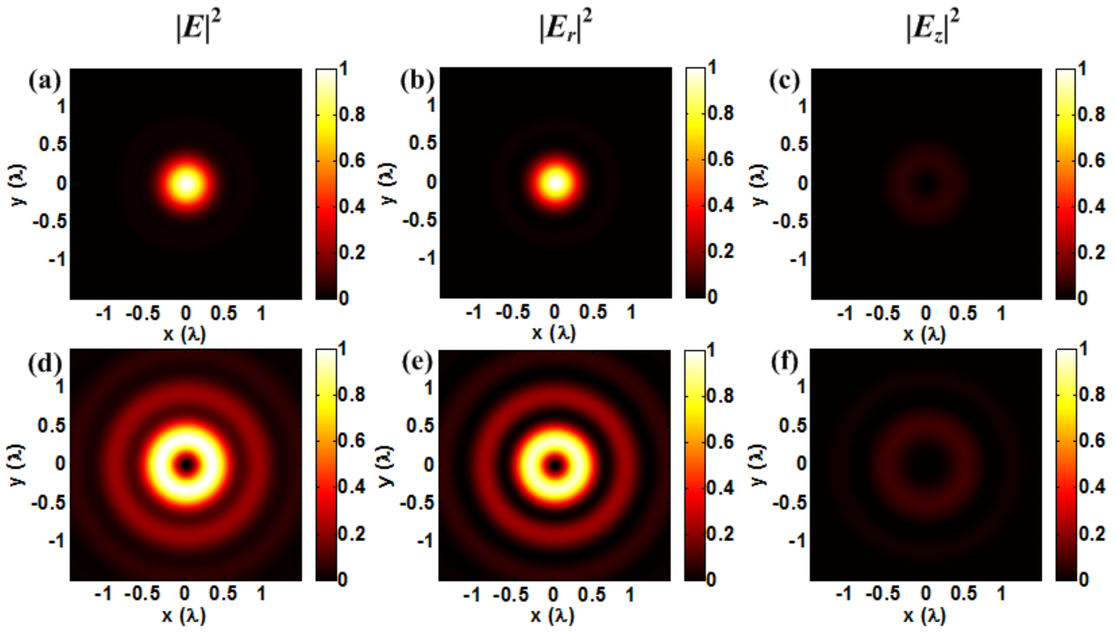

Fig. S2 Comparisons of the intensity distributions of the total field $|E|^2$ ((a) and (c)), transversal component $|E_r|^2$ ((b) and (e)) and longitudinal component $|E_z|^2$ ((c) and (f)) of the bright focal spot ((a)-(c)) formed by focusing the circularly polarized beam and the dark focal spot ((d)-(f)) formed by focusing the circularly polarized vortex beam. The intensity is normalized to the maximum total intensity respectively. The calculation was done with a high-NA water-immersion objective (1.1 NA, 60×).

Fig. S2 compares the intensity distributions of the bright and dark focal spot formed by focusing circularly polarized and circular polarized vortex beams, respectively, including the transversal and longitudinal components. Longitudinal fields occur when the above beams are focused by a high-NA objective lens. However, the transversal components still dominate in the focal fields. The longitudinal components accounts for only a small portion of the total intensity, less than 6% of the total intensity for the bright focal field and almost 10% for the dark focal field. Considering the intensity-squared dependence of SHG excitation, the longitudinal

components will contribute less in the excitation.

S2. Effects of the subtractive factor on the imaging

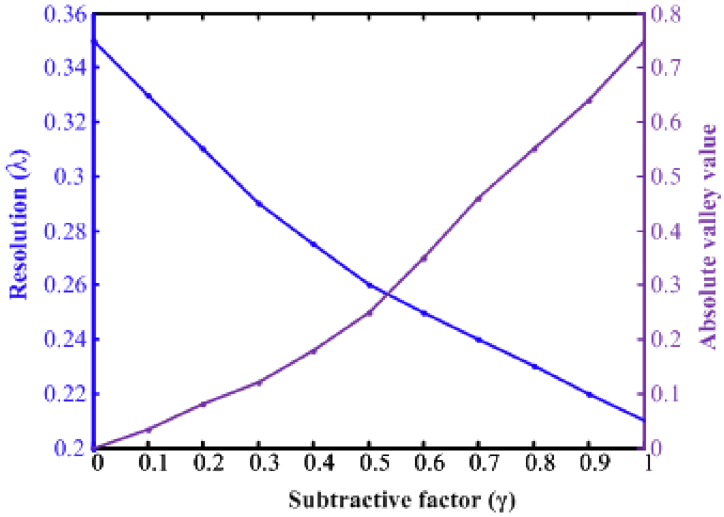

Fig. S3 Simulated resolution (blue line) and absolute value of the minimum value (red line) in the subtraction under different subtractive factors.

Fig. S3 shows the relations between the subtractive factor γ and the resolution enhancement as well as the negative values in the subtractive process. If we increase the factor γ, the resolution and contrast will be further enhanced. However, the subtraction process will inevitably produces negative values around the objective since the intensity distributions of the two focal spots are not ideally matched. This has no effects on the imaging if the sample is sparse. But when we image high-density samples, large negative values caused by the high γ value will deteriorate the quality of information. A compromise should be made between the resolution enhancement and image quality according to the experiments. When the γ factor is larger than 0.5, the resolution enhancement is not so evident while the absolute value of the minimum negative value increases dramatically. Thus a γ factor around 0.5 is optimal to achieve modest resolution enhancement and at the same time ensure the image quality in our

experiments.

S3. Experimental setup

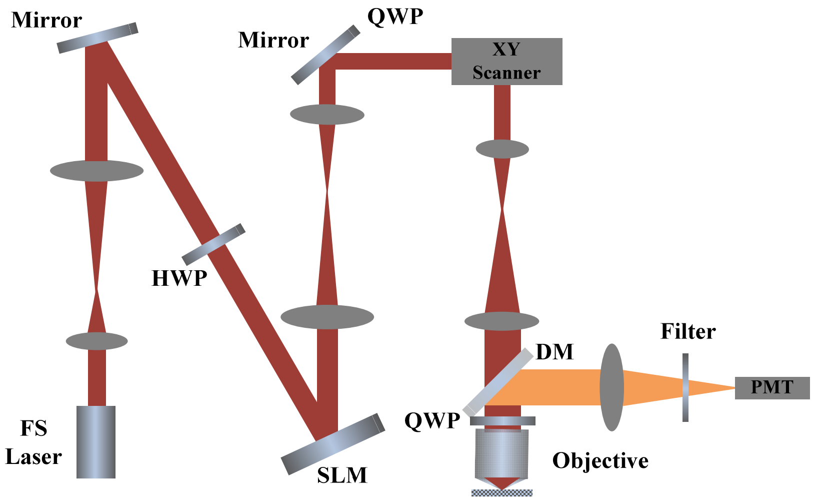

Fig. S4 Experimental setup for subtractive SHG imaging using an SLM. HWP, half wavelength plate; QWP, quarter wavelength plate; DM, diachronic mirror.

S4. Experimental formation of the bright and dark beams

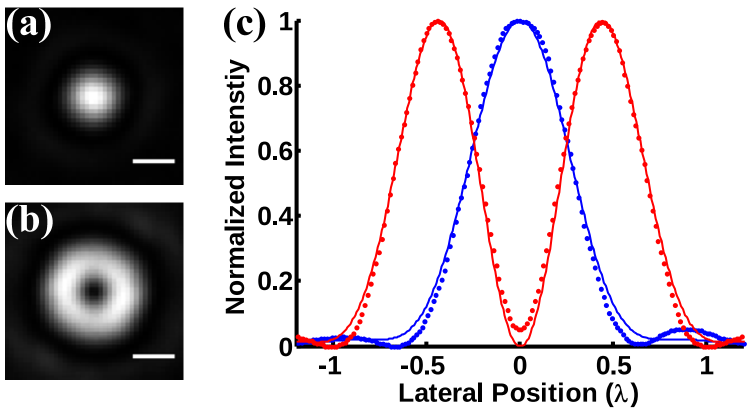

Fig. S5 (a)-(b) Experimental characterization of the intensity distributions in the focal plane of the (a) bright and (b) dark focal spot. (c) Theoretical (solid lines) and experimental (dotted lines) intensity profiles of the bright (blue) and dark (red) beams. Scale bar: 500 nm

S5. Assessment of resolution enhancement

Table S1 Resolution measurements of different NPs (nm)

Number	1	2	3	4	5
Conventional imaging	278	284	280	292	274
Subtractive imaging	207	215	212	218	207
Enhancement	25.5%	24.3%	24.6%	25.3%	24.5%

Table S1 Resolution measurements of different tendons (nm)

Number	1	2	3	4	5
Conventional imaging	273	290	282	278	284
Subtractive imaging	206	215	214	208	216
Enhancement	24.5%	25.8%	24.1%	25.1%	23.9%

S6. Images of the fibrils with different orientations

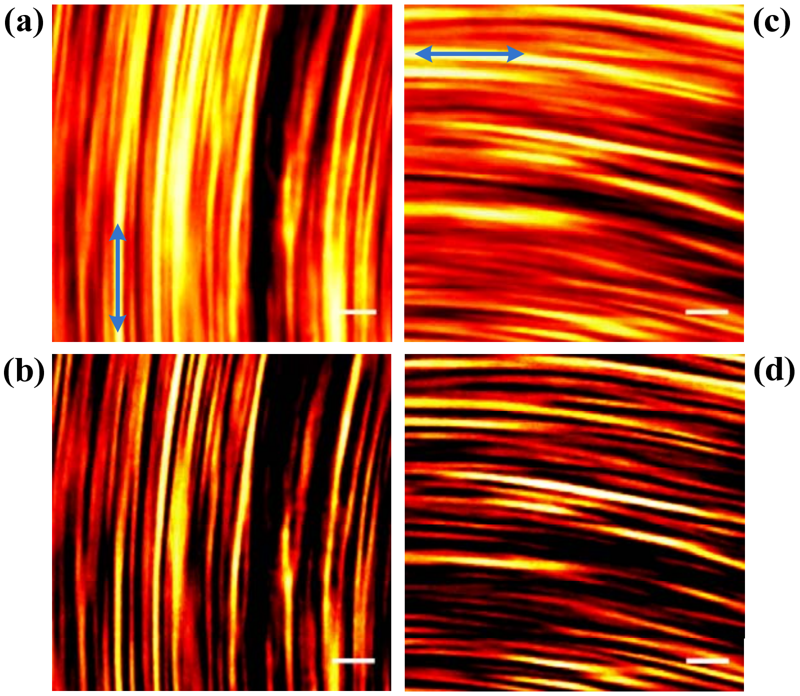

Fig. S6 Conventional ((a) and (c)) and subtractive SHG images ((b) and (d)) of the fibrils with different orientations. The blue arrow denotes the collagen fibril longitudinal axis. Scale bar: 2 μm.

Reference

1. Youngworth, K. & Brown, T. Focusing of high numerical aperture cylindrical-vector beams. *Opt. Express* **7,** 77–87 (2000).
2. Zhan, Q. Properties of circularly polarized vortex beams. *Opt. Lett.* **31,** 867–869 (2006).
